# Lenvatinib plus Pembrolizumab for Patients with Previously Treated Advanced Gastric, Biliary Tract, or Pancreatic Cancer: Results from the Phase II LEAP-005 Study

**DOI:** 10.1158/2767-9764.CRC-26-0018

**Published:** 2026-03-26

**Authors:** Mariano Ponz-Sarvisé, Sun Young Rha, Carlos A. Gomez-Roca, Laura Ortega Morán, Sanjeev Gill, Giampaolo Tortora, Ravit Geva, Esma Saada-Bouzid, Armando Santoro, Tae Won Kim, Daniel Heudobler, Corina E. Dutcus, Chinyere E. Okpara, Razi Ghori, Yiwei Zhang, Amir Vajdi, E.J. Dettman, Fan Jin, Roman Groisberg, Ronnie Shapira-Frommer

**Affiliations:** 1 Department of Medical Oncology and Program in Solid Tumors, Cancer Center Clínica Universidad de Navarra (CCUN), Cima-Universidad de Navarra, Pamplona, Spain.; 2 Yonsei Cancer Center, Yonsei University College of Medicine, Seoul, Republic of Korea.; 3 Institut Universitaire du Cancer de Toulouse Oncopole, Toulouse, France.; 4 Department of Medical Oncology, Hospital General Universitario Gregorio Marañón, Madrid, Spain.; 5 The Alfred Hospital, Melbourne, Australia.; 6 Medical Oncology, Fondazione Policlinico A Gemelli IRCCS, Rome, Italy.; 7 Medical Oncology, Università Cattolica del Sacro Cuore, Rome, Italy.; 8 Tel Aviv Sourasky Medical Center, Tel Aviv, Israel.; 9 Early Phase Trial Unit, Centre Antoine Lacassagne, Nice, France.; 10 Laboratory of Translational Research, Côte d’Azur University, Nice, France.; 11 IRCCS, Humanitas Research Hospital, Milan, Italy.; 12 Department of Biomedical Sciences, Humanitas University, Milan, Italy.; 13 Department of Oncology, Asan Medical Center, University of Ulsan, Seoul, Republic of Korea.; 14 University Hospital Regensburg, Regensburg, Germany.; 15 Eisai Inc., Nutley, New Jersey.; 16 Eisai Ltd., Hatfield, United Kingdom.; 17 Merck & Co., Inc., Rahway, New Jersey.; 18 Ella Lemelbaum Institute for Immuno-Oncology, Sheba Medical Center, Ramat Gan, Israel.

## Abstract

**Purpose:**

Patients with gastric cancer, biliary tract cancer (BTC), and pancreatic ductal adenocarcinoma (PDAC) have poor survival outcomes and limited second- or later-line treatment options. Certain drugs targeting vascular endothelial growth factor (VEGF) or programmed cell death protein 1 (PD-1) signaling pathways are currently used in these cancers in specific circumstances; however, there remains a need for novel treatment combinations. LEAP-005 is a multicohort, open-label, phase II study that evaluated lenvatinib (multitargeted inhibitor of tyrosine kinases, including VEGF) plus pembrolizumab (anti–PD-1 monoclonal antibody) in select previously treated solid tumors.

**Patients and Methods:**

Participants with previously treated, advanced gastric cancer, BTC, and PDAC were enrolled in cohorts C, F, and G of LEAP-005, respectively, and received lenvatinib 20 mg/day orally plus pembrolizumab 200 mg i.v. every 3 weeks. Primary endpoints were objective response rate (ORR) and safety.

**Results:**

Of 99, 102, and 103 total participants enrolled in cohorts C, F, and G, respectively, median times from first dose of study treatment to data cutoff (February 6, 2023) were 23.7, 24.2, and 19.5 months. ORRs (95% confidence interval) by blinded independent central review were 15.2% (8.7%–23.8%) in cohort C, 17.6% (10.8%–26.4%) in cohort F, and 7.8% (3.4%–14.7%) in cohort G. Grade 3 to 5 treatment-related adverse events occurred in 54.5% of participants in cohort C, and grade 3 to 4 (no grade 5) occurred in 60.8% and 59.2% of participants in cohorts F and G, respectively.

**Conclusions:**

Lenvatinib plus pembrolizumab demonstrated modest antitumor activity and a manageable safety profile in previously treated, advanced gastric cancer, BTC, and PDAC.

**Significance:**

In the phase II LEAP-005 study, lenvatinib plus pembrolizumab showed modest antitumor activity and a manageable safety profile in participants with previously treated gastrointestinal-related cancers. Exploratory analyses in participants with BTC indicated higher ORRs in participants with targetable alterations versus those without.

## Introduction

Patients with gastric cancer, biliary tract cancer (BTC), and pancreatic ductal adenocarcinoma (PDAC) have poor survival outcomes (1–3). First-line treatment options for advanced stages of these cancers include different chemotherapy-based regimens, some of which incorporate targeted therapy or immunotherapy, and for certain patients, biomarker-directed targeted therapy or immunotherapy; however, second-line or later treatment options are limited (4–6).

Vascular endothelial growth factor (VEGF) is an important driver of tumor angiogenesis and a modulator of the tumor microenvironment (7). VEGF signaling also plays a key role in regulating T-cell activation and has been found to increase the expression of several immune checkpoint proteins, including programmed cell death protein 1 (PD-1), leading to an immunosuppressive tumor microenvironment (8). The binding of PD-1 to its ligands, programmed cell death ligand 1 (PD-L1) or programmed cell death ligand 2 (PD-L2), can inhibit effector T-cell responses and thereby promote tumor escape (8). Certain agents targeting VEGF or PD-1 signaling pathways are indicated in patients with gastric cancer, BTC, or PDAC in certain circumstances (4–6).

Pembrolizumab, an anti–PD-1 monoclonal antibody, is a standard-of-care treatment as monotherapy or in combination with other therapies in multiple tumor types (9), including in combination with trastuzumab and chemotherapy as first-line therapy in human epidermal growth factor receptor 2 (HER2)–positive gastric or gastroesophageal junction (GEJ) cancer (10). Pembrolizumab monotherapy has demonstrated modest antitumor activity in gastric cancer (11). Pembrolizumab plus chemotherapy improved overall survival (OS) over chemotherapy alone in participants with previously untreated metastatic or unresectable BTC (12), leading to regulatory approval by the US FDA in this setting. There is limited evidence of efficacy for anti–PD-1/–PD-L1 [anti–PD-(L)1] agents as monotherapy in pancreatic cancer (13, 14). Pembrolizumab combined with chemotherapy has demonstrated modest antitumor activity in metastatic PDAC (15). Furthermore, pembrolizumab plus chemoradiation has shown promising recurrence-free survival as a neoadjuvant regimen in participants with resectable or borderline resectable pancreatic cancer (16). Tissue-based biomarkers may also be important for pembrolizumab as analyses from the KEYNOTE-028 and KEYNOTE-158 studies demonstrated that the T cell–inflamed gene expression profile (Tcell_inf_GEP), PD-L1 expression, and tumor mutational burden (TMB) were associated with response to pembrolizumab monotherapy across a range of advanced solid tumors (17, 18).

Lenvatinib, a multikinase inhibitor of VEGF receptors 1 to 3 and other receptor tyrosine kinases, including fibroblast growth factor receptors (FGFR) 1 to 4, platelet-derived growth factor α, KIT, and RET, is indicated in the treatment of several tumor types as monotherapy and in combination with other immunotherapies (19). Inhibition of tyrosine kinase activity by lenvatinib promotes a shift in the tumor microenvironment to an immune-stimulatory state (20). Lenvatinib monotherapy demonstrated antitumor activity in participants with advanced BTC who had received one prior line of therapy (21). Lenvatinib combined with pembrolizumab has the potential to improve antitumor activity over single-agent treatment (8, 22). In preclinical models, lenvatinib plus an anti–PD-1 monoclonal antibody led to immunomodulatory effects, including increases in CD8^+^ T cells and depletion of monocytes and macrophages (23), significantly inhibited *in vivo* tumor growth of CT26 isografts, and suppressed tumor growth compared with either treatment alone (24). Preclinical data have also suggested that inhibition of FGFR by lenvatinib reactivates IFNγ signaling pathways, leading to upregulation of PD-L1 expression in tumors, thereby enhancing the antitumor activity of lenvatinib combined with anti–PD-1 antibodies (20). Subsequent clinical trials have demonstrated the antitumor activity and manageable safety profile of lenvatinib plus pembrolizumab in select advanced solid tumors (25–30).

LEAP-005 (ClinicalTrials.gov, NCT03797326) is a multicohort, open-label, phase II study that evaluated the antitumor activity and safety of lenvatinib plus pembrolizumab in participants with selected previously treated solid tumors. Additionally, we conducted an exploratory analysis of the association between clinical outcomes and PD-L1 status, TMB, and Tcell_inf_GEP to identify participants more likely to respond to lenvatinib plus pembrolizumab.

## Patients and Methods

### Eligibility criteria

This report includes participants with gastric cancer (cohort C), BTC (cohort F), and PDAC (cohort G) enrolled in the LEAP-005 study. Participants aged ≥18 years were eligible if they had a histologically or cytologically documented advanced (metastatic and/or unresectable) solid tumor that was incurable and for which prior standard systemic therapy had failed, had disease progression (PD) on or since the last treatment, had measurable disease per Response Evaluation Criteria in Solid Tumors (RECIST; RRID: SCR_026435) version 1.1 as assessed by the investigator and confirmed by blinded independent central review (BICR), and had provided a PD-L1–evaluable archival tumor tissue sample or newly obtained core or excisional biopsy of a tumor lesion not previously irradiated.

Eligible participants for cohort C had gastric or GEJ adenocarcinoma (squamous cell carcinoma was excluded) and had received two prior lines of therapy (prior neoadjuvant or adjuvant systemic cytotoxic chemotherapy used in the initial treatment was not considered a prior line of therapy unless completed ≤12 months before the current tumor recurrence). Eligible participants for cohort F had BTC and had received one prior line of therapy (prior neoadjuvant or adjuvant systemic cytotoxic chemotherapy used in the initial treatment was not considered a prior line of therapy unless completed ≤6 months before the current tumor recurrence) and a Child–Pugh score of 5 or 6 (class A: well-compensated disease). Eligible participants for cohort G had pathologically (histologically or cytologically) confirmed metastatic PDAC and had received one or two lines of prior therapy, of which at least one was a platinum- or gemcitabine-containing regimen. For each cohort, all systemic cytotoxic chemotherapies, including antibody–drug conjugates with a cytotoxic warhead, were considered prior lines of therapy.

Other inclusion criteria for all cohorts included an Eastern Cooperative Oncology Group (ECOG) performance status (RRID: SCR_026432) of 0 or 1, adequate organ function per protocol specifications, adequately controlled blood pressure with or without antihypertensive medications (defined as ≤150/90 mm Hg), and life expectancy of ≥12 weeks.

Exclusion criteria, across all cohorts, included radiographic evidence of major blood vessel invasion or infiltration; clinically significant hemoptysis or tumor bleeding ≤2 weeks before the first dose of study drug; significant cardiovascular impairment or history of arterial thromboembolism ≤12 months before the first dose; serious nonhealing wound, ulcer, or bone fracture; major surgery ≤3 weeks before first dose; receipt of biologic response modifiers ≤4 weeks before study entry; prior therapy with lenvatinib, an anti–PD-(L)1 or anti–PD-L2 agent, or any agent directed to another stimulatory or coinhibitory T-cell receptor; preexisting grade ≥3 fistula; urine protein ≥1 g/24 hours; QTc prolongation >480 milliseconds or left ventricular ejection fraction <55%; diagnosis of immunodeficiency or receipt of chronic systemic steroid therapy or immunosuppressive therapy ≤7 days before the first dose; known active central nervous system metastases or carcinomatous meningitis; tumors involving the brainstem; active autoimmune disease that required treatment ≤2 years before the study began; history of or current pneumonitis; and active infection requiring systemic therapy.

### Study design

LEAP-005 was a multicohort, multicenter, open-label, phase II study. Cohorts C, F, and G enrolled participants with gastrointestinal-related cancers. Participants received lenvatinib 20 mg/day orally plus pembrolizumab (RRID: AB_3076193) 200 mg i.v. every 3 weeks. Treatment continued for up to 35 cycles (pembrolizumab only) or until PD, unacceptable toxicity, initiation of new anticancer treatment, or participant withdrawal. Pembrolizumab treatment beyond PD per RECIST version 1.1 was permitted per investigator discretion. Treatment with lenvatinib could continue beyond 2 years if participants experienced treatment benefit per investigator discretion and in consultation with the sponsor. Participants who discontinued treatment because of adverse events (AE) could discontinue one or both study drugs depending on which drugs were deemed to be related to the AE per investigator assessment.

The study was conducted in accordance with the ethical principles of the Declaration of Helsinki that are consistent with Good Clinical Practice and was approved by the appropriate institutional review boards and regulatory agencies. All participants provided written informed consent before participation.

### Assessments

Tumor PD-L1 status was assessed centrally using the PD-L1 IHC 22C3 pharmDx (investigational use only) diagnostic kit (Agilent Technologies). Combined positive score (CPS) was calculated as the number of PD-L1–staining cells (tumor cells, lymphocytes, and macrophages) divided by the total number of viable tumor cells, multiplied by 100. CPS raw scores were interpreted prospectively at the time the samples were evaluated at the testing laboratory, and PD-L1 status per CPS was applied retrospectively based on these raw scores. Based on prior evidence suggesting that immunotherapy may have greater benefit with higher PD-L1 CPS in gastric cancer (31), the CPS threshold was set at CPS ≥10 and <10 in cohort C (gastric cancer) and was set at CPS ≥1 and <1 in cohorts F (BTC) and G (PDAC; ref. 32).

Tumor imaging was performed at baseline, every 9 weeks from the time of treatment initiation for 54 weeks, then every 12 weeks through week 102, and every 24 weeks thereafter. AEs were monitored throughout study treatment and for 30 days after the last dose of study drug (90 days for serious AEs) and were graded according to NCI Common Terminology Criteria for Adverse Events version 4.0 (RRID: SCR_010296).

### Endpoints

The primary endpoints were objective response rate [ORR; proportion of participants with best overall response of complete response (CR) or partial response (PR)] per RECIST version 1.1 by investigator assessment and by BICR in cohorts for which the final target enrollment was 100 participants (see description of population size and cohort expansion below) and safety (AEs and discontinuations due to AEs). Secondary endpoints were disease control rate (DCR; proportion of participants with best overall response of CR, PR, or stable disease), duration of response (DOR; time from earliest evidence of CR or PR until PD or death due to any cause), progression-free survival (PFS; time from first dose of study drug until PD or death from any cause), and OS (time from first dose of study drug until death from any cause).

### Exploratory biomarker analyses

Further retrospective exploratory biomarker analyses for cohort F (BTC) were performed to better understand response in this tumor type, which has several biomarker-directed treatment options (5). Formalin-fixed, paraffin-embedded tumor tissue samples were used, with whole-exome sequencing (WES) performed using next-generation sequencing and RNA sequencing analyses performed using TruSeq on the NovaSeq platform (RRID: SCR_016387, Illumina). TMB status [high TMB (TMB-H) defined as ≥175 mutations/exome, corresponding with ≥10 mutations/megabase; ref. 33], microsatellite instability (MSI) status [high MSI (MSI-H) defined as instability of >20% of microsatellite loci as detected per mSINGS (RRID: SCR_027728)], 18-gene Tcell_inf_GEP, and 11 other non-GEP signatures (*RAS*, angiogenesis, monocytic myeloid-derived suppressor cells, granulocytic myeloid-derived suppressor cells, glycolysis, hypoxia, *MYC*, proliferation, stromal/epithelial–mesenchymal transition/TGFβ, WNT, and MVD) were determined as previously described (33–36). Further details on WES analysis methodology are summarized in Supplementary Fig. S1 (33, 36).

### Statistical analysis

For cohorts C and F, 30 participants were enrolled initially in each cohort. Based on an interim analysis of efficacy per investigator assessment conducted 6 months after the final participant was enrolled, up to an additional 70 participants could be enrolled in each cohort per sponsor review of results. Cohort G was planned to enroll 100 participants.

Efficacy and safety were assessed in all participants who received ≥1 dose of pembrolizumab plus lenvatinib. Point estimates and exact 95% Clopper–Pearson confidence intervals (CI) were provided for ORR and DCR. DOR, PFS, and OS were estimated by the Kaplan–Meier method. Statistical analyses were performed using SAS version 9.4 (RRID: SCR_008567, SAS Institute, Inc.).

Exploratory biomarker analyses were performed retrospectively in cohort F according to a separate statistical analysis plan. Associations of RNA-based biomarkers, including Tcell_inf_GEP and other RNA gene signatures, with clinical outcomes were assessed using logistic regression (ORR) and Cox proportional hazards models (PFS and OS) adjusted for ECOG performance status. The 95% CIs for the best overall response were estimated using the Clopper and Pearson method. The discriminatory value of the RNA gene signatures to ORR was evaluated using area under the receiver operating characteristic (AUROC) curve analysis. For non-Tcell_inf_GEP RNA gene signatures, *P* values were adjusted for multiplicity using the Hochberg step-up procedure with significance level prespecified at α = 0.1. The same method was applied to non-Tcell_inf_GEP RNA gene signatures with Tcell_inf_GEP adjustment, evaluating any additional explanatory value beyond Tcell_inf_GEP. Descriptive analyses were performed to assess the association of gene mutations, including select genes related to lenvatinib (*FGFR1–4*), and TMB with clinical outcomes.

## Results

### Study population

**Figure 1.**
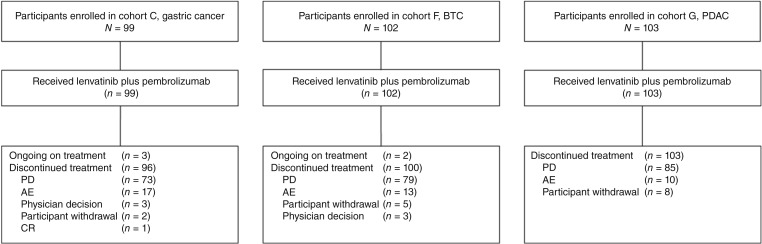
Participant disposition in cohorts C, gastric cancer; F, BTC; and G, PDAC.

Participants were enrolled in cohort C (gastric cancer) between March 24, 2019, and October 11, 2021; in cohort F (BTC) between April 1, 2019, and October 4, 2021; and in cohort G (PDAC) between March 24, 2021, and September 2, 2021. After enrolling an initial allocation of 30 participants each in cohorts C and F, interim analysis expansion criteria for these cohorts were met; total enrollment was 99 participants in cohort C and 102 participants in cohort F. Cohort G did not include an expansion phase and enrolled a total of 103 participants. At the time of analysis, all participants had discontinued study treatment except for three participants in cohort C and two in cohort F (Fig. 1). The median (range) times from first dose to the data cutoff date of February 6, 2023, were 23.7 (15.2–45.8) months in cohort C, 24.2 (14.9–45.8) months in cohort F, and 19.5 (15.8–21.8) months in cohort G. The median (range) durations of treatment with lenvatinib plus pembrolizumab were 2.3 (0–22.7) months in cohort C, 3.6 (0.1–31.5) months in cohort F, and 2.1 (0.2–12.5) months in cohort G. The median (range) number of doses of lenvatinib was 64 (1–690) and of pembrolizumab was four (1–33) in cohort C; 83.5 (2–882) and five (1–35), respectively, in cohort F; and 63 (5–343) and four (1–17), respectively, in cohort G. Baseline demographics and clinical characteristics for each cohort are provided in Table 1. Representativeness of the study population is shown in Supplementary Table S1.

**Table 1.** Baseline demographics and disease characteristics.

​	Gastric cancer (cohort C) *N* = 99	BTC (cohort F) *N* = 102	PDAC (cohort G) *N* = 103
Age, median (range), years	60 (28–83)	64.5 (31–80)	63 (34–83)
Male	69 (69.7)	50 (49)	61 (59.2)
Race	​	​	​
Asian	11 (11.1)	18 (17.6)	8 (7.8)
Black or African American	2 (2)	1 (1)	2 (1.9)
Multiple	2 (2)	0	3 (2.9)
White	69 (69.7)	70 (68.6)	90 (87.4)
Not reported	15 (15.2)	13 (12.7)	0
ECOG performance status	​	​	​
0	34 (34.3)	47 (46.1)	40 (38.8)
1	65 (65.7)	55 (53.9)	63 (61.2)
Metastatic stage	​	​	​
M0	1 (1)	7 (6.9)	2 (1.9)
M1	98 (99)	95 (93.1)	101 (98.1)
No. of prior lines of systemic therapy	​	​	​
1	NA	95 (93.1)	39 (37.9)
2	91 (91.9)	6 (5.9)	64 (62.1)
3	7 (7.1)	1 (1)	0
4	1 (1)	0	0
PD-L1 status	​	​	​
Positive	49 (49.5)^a^	63 (61.8)^b^	60 (58.3)^b^
Negative	46 (46.5)^c^	35 (34.3)^d^	36 (35)^d^
Missing	4 (4)	4 (3.9)	7 (6.8)
MSI status^e^	​	​	​
MSI-H	2 (2)	0	2 (1.9)
MSI-L	3 (3)	0	6 (5.8)
MSS	10 (10.1)	0	21 (20.4)
Missing	84 (84.8)	102 (100)^f^	74 (71.8)
Prior bevacizumab use	0	0	0

Unless specified otherwise, data are *n* (%).

Abbreviations: MSI-L, microsatellite instability low; MSS, microsatellite stable; NA, not applicable.

a PD-L1 CPS ≥10.

b PD-L1 CPS ≥1.

c PD-L1 CPS <10.

d PD-L1 CPS <1.

e As determined per local testing.

f Based on WES, per central testing: MSI-H, *n* = 1.

### Efficacy

#### Cohort C, gastric cancer

**Figure 2.**
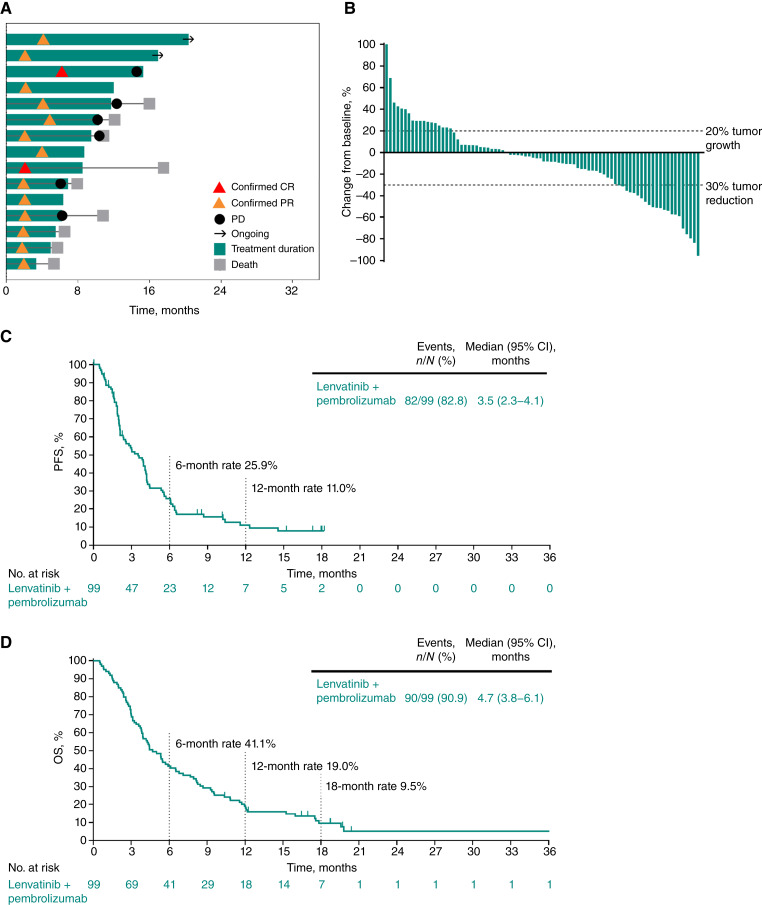
Efficacy results in cohort C, gastric cancer. **A,** Time on study treatment and treatment response per RECIST version 1.1 by BICR for participants with an objective response (confirmed CR or PR). **B**, Best percentage change from baseline in target lesion size per RECIST version 1.1 by BICR among participants with ≥1 postbaseline assessment. **C**, PFS per RECIST version 1.1 by BICR. **D**, OS. In **B**, percentage changes from baseline >100% are presented as 100%.

The ORR (95% CI) per RECIST version 1.1 by BICR in cohort C was 15.2% (8.7%–23.8%); two participants (2%) had a CR and 13 (13.1%) had a PR (Table 2). The DCR (95% CI) was 53.5% (43.2%–63.6%). ORR was higher in participants with tumor PD-L1 CPS ≥10 than in those with CPS <10. In the 15 participants with a response, the median (range) DOR was 8.3 (3.4 to 16.2+) months (Table 2; Fig. 2A). Of 84 participants with ≥1 postbaseline tumor assessment, 51 (60.7%) had a reduction from baseline in tumor size as the best percentage change from baseline in target lesion size (Fig. 2B).

**Table 2.** Antitumor activity per RECIST version 1.1 by BICR.

​	Gastric cancer (cohort C)			BTC (cohort F)			PDAC (cohort G)		
	All participants (*N* = 99)^a^	Tumor PD-L1 CPS ≥10 (*n* = 49)	Tumor PD-L1 CPS <10 (*n* = 46)	All participants (*N* = 102)^a^	Tumor PD-L1 CPS ≥1 (*n* = 63)	Tumor PD-L1 CPS <1 (*n* = 35)	All participants (*N* = 103)^b^	Tumor PD-L1 CPS ≥1 (*n* = 60)	Tumor PD-L1 CPS <1 (*n* = 36)
ORR (95% CI), %	15.2 (8.7–23.8)	22.4 (11.8–36.6)	8.7 (2.4–20.8)	17.6 (10.8–26.4)	19 (10.2–30.9)	14.3 (4.8–30.3)	7.8 (3.4–14.7)	5 (1–13.9)	11.1 (3.1–26.1)
Best overall response, *n* (%)	​	​	​	​	​	​	​	​	​
CR	2 (2)	0	2 (4.3)	4 (3.9)	2 (3.2)	2 (5.7)	0	0	0
PR	13 (13.1)	11 (22.4)	2 (4.3)	14 (13.7)	10 (15.9)	3 (8.6)	8 (7.8)	3 (5)	4 (11.1)
SD	38 (38.4)	15 (30.6)	21 (45.7)	48 (47.1)	31 (49.2)	16 (45.7)	31 (30.1)	14 (23.3)	15 (41.7)
PD	28 (28.3)	15 (30.6)	13 (28.3)	28 (27.5)	16 (25.4)	10 (28.6)	47 (45.6)	31 (51.7)	13 (36.1)
Not evaluable^c^	4 (4)	2 (4.1)	2 (4.3)	4 (3.9)	2 (3.2)	2 (5.7)	2 (1.9)	1 (1.7)	1 (2.8)
No assessment^d^	14 (14.1)	6 (12.2)	6 (13)	4 (3.9)	2 (3.2)	2 (5.7)	15 (14.6)	11 (18.3)	3 (8.3)
DCR, *n* (%)	53 (53.5)	26 (53.1)	25 (54.3)	66 (64.7)	43 (68.3)	21 (60)	39 (37.9)	17 (28.3)	19 (52.8)
Median DOR (range), months	8.3 (3.4 to 16.2+)	8.3 (3.4 to 16.2+)	8.2 (4–8.4)	6.2 (2.7 to 19.6+)	6.4 (3.3+ to 19.6+)	4.2 (2.7 to 18.9+)	5.8 (2.1+ to 8.6)	8.6 (2.7–8.6)	4.9 (4.2–5.8)

Abbreviation: SD, stable disease.

“+” indicates that there was no PD at the time of last disease assessment.

a PD-L1 status was not available for four participants.

b PD-L1 status was not available for seven participants.

c “Not evaluable” includes participants with postbaseline imaging whose best overall response was determined to be not evaluable per RECIST version 1.1.

d “No assessment” includes participants who had a baseline assessment but no postbaseline assessment.

At data cutoff, 82 participants (82.8%) in cohort C had experienced a PFS event. The median (95% CI) PFS was 3.5 (2.3–4.1) months, and PFS rates were 25.9% at 6 months and 11% at 12 months (Fig. 2C). At data cutoff, 90 participants (90.9%) had died. The median (95% CI) OS was 4.7 (3.8–6.1) months, and OS rates were 41.4%, 19%, and 9.5% at 6, 12, and 18 months, respectively (Fig. 2D).

Ninety-seven participants in cohort C had tumors classified as non–MSI-H per local testing [84 of these participants (86.6%) had missing data]. The ORR and DOR in participants whose tumors were classified as non–MSI-H are provided in Supplementary Table S2. PFS and OS by baseline PD-L1 status are provided in Supplementary Fig. S2A and S2B.

#### Cohort F, BTC

**Figure 3.**
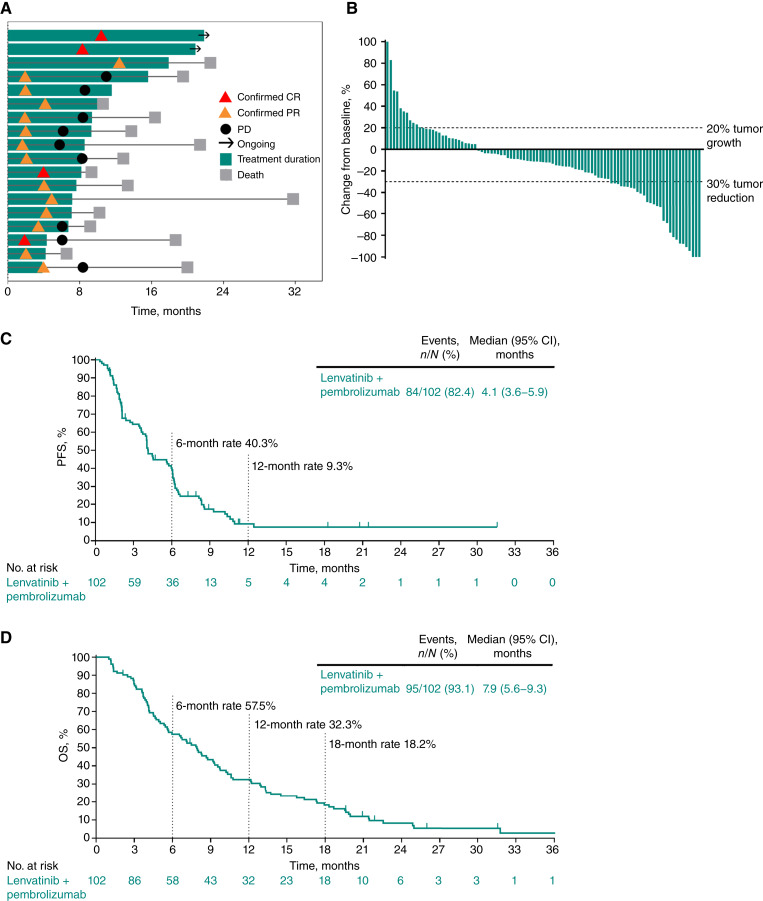
Efficacy results in cohort F, BTC. **A,** Time on study treatment and treatment response per RECIST version 1.1 by BICR for participants with an objective response (confirmed CR or PR). **B**, Best percentage change from baseline in target lesion size per RECIST version 1.1 by BICR among participants with ≥1 postbaseline assessment. **C**, PFS per RECIST version 1.1 by BICR. **D**, OS. In **B**, percentage changes from baseline >100% are presented as 100%.

The ORR (95% CI) per RECIST version 1.1 by BICR in cohort F was 17.6% (10.8%–26.4%); four participants (3.9%) had a CR and 14 (13.7%) had a PR (Table 2). The DCR (95% CI) was 64.7% (54.6%–73.9%). ORR was similar regardless of tumor PD-L1 CPS. In the 18 participants with a response, the median (range) DOR was 6.2 (2.7 to 19.6+) months (Table 2; Fig. 3A). In the 97 participants with ≥1 postbaseline tumor assessment, 69 (71.1%) had a reduction in tumor size as the best percentage change from baseline in target lesion size relative to baseline (Fig. 3B).

At data cutoff, 84 participants (82.4%) in cohort F had experienced a PFS event. The median (95% CI) PFS was 4.1 (3.6–5.9) months, with PFS rates of 40.3% at 6 months and 9.3% at 12 months (Fig. 3C). Ninety-five participants (93.1%) had died at the time of data cutoff. The median (95% CI) OS was 7.9 (5.6–9.3) months, with OS rates of 57.5%, 32.3%, and 18.2% at 6, 12, and 18 months, respectively (Fig. 3D).

All 102 participants in cohort F had missing MSI status at baseline, per local testing; these tumors were classified as non–MSI-H (Supplementary Table S2). PFS and OS by baseline PD-L1 status are provided in Supplementary Fig. S3A and S3B.

Among participants in cohort F, WES data were available for 83 participants (Supplementary Fig. S1), with the following biomarkers identified: *IDH1* mutation in *n* = 9 (10.8%); *NTRK1/3* mutations in *n* = 4 (4.8%); TMB-H in *n* = 3 (3.6%); *FGFR2* mutation in *n* = 6 (7.2%), including *FGFR2* fusion in *n* = 1 (1.2%); *HER2* amplification in *n* = 1 (1.2%); and MSI-H in *n* = 1 (1.2%; one participant with missing MSI status at baseline, per local testing, had MSI-H status by WES, per central testing). ORR (95% CI) among these 83 participants was 15.7% (8.6%–25.3%) and among those without available data (*n* = 19) was 26.3% (9.1%–51.2%; Supplementary Table S3). There was a trend for higher ORR and longer PFS and OS in participants whose tumors were *TP53* wild type (*n* = 48) than in those with *TP53*-mutant tumors (*n* = 35; Supplementary Table S3; Supplementary Fig. S4A and S4B). The same trend was observed in participants with *KRAS*–wild-type tumors (*n* = 69) versus those with *KRAS*-mutant tumors (*n* = 14; Supplementary Table S3; Supplementary Fig. S5A and S5B). Small numbers of participants with other individual targetable alterations prohibited further analyses in individual groups; however, combining those with tumors with *FGFR2* fusions, *HER2* amplifications, *IDH1* mutations, *NTRK* fusions, TMB-H, or MSI-H status, there was a trend of higher ORR among those with (*n* = 14) versus without (*n* = 69) these alterations (Supplementary Table S3).

The median TMB scores were similar for responders (*n* = 13) and nonresponders (*n* = 70; Supplementary Fig. S6A and S6B), with few participants having TMB-H tumors (*n* = 3, as noted above). The AUROC analysis did not indicate an association between TMB and achieving objective response [area under the curve (AUC), 0.55 (95% CI, 0.39–0.71)].

Seventy-four participants in cohort F had evaluable data from RNA sequencing analysis. There were no associations between Tcell_inf_GEP and ORR, PFS, or OS (Supplementary Tables S3 and S4; Supplementary Fig. S7A and S7B). The median Tcell_inf_GEP scores were similar for treatment responders (*n* = 13) and nonresponders (*n* = 61), and AUROC analysis did not indicate an association between Tcell_inf_GEP score and achieving objective response [AUC (95% CI), 0.53 (0.36–0.70); Supplementary Fig. S8A and S8B]. Analysis of other non-Tcell_inf_GEP signatures showed a significant negative association between *RAS* signature and PFS after adjustment for multiplicity and regardless of adjustment for Tcell_inf_GEP (*P* = 0.062 without Tcell_inf_GEP adjustment; *P* = 0.069 with Tcell_inf_GEP adjustment; Supplementary Table S4). Additionally, the median *RAS* signature score was lower in responders than in nonresponders, and the AUROC analysis indicated a negative association between *RAS* and achieving an objective response [AUC (95% CI), 0.63 (0.46–0.79); Supplementary Fig. S9A and S9B]. *RAS* signature scores were higher in participants with *KRAS*-mutant tumors than in those with *KRAS*–wild-type tumors (Supplementary Fig. S10).

In the analysis of select additional genes related to lenvatinib, *FGFR3* and *FGFR4* gene expression showed a positive trend of association with achieving an objective response [AUC (95% CI), 0.59 (0.41–0.78) for *FGFR3*; 0.64 (0.48–0.80) for *FGFR4*; Supplementary Fig. S11A–S11D].

#### Cohort G, PDAC

**Figure 4.**
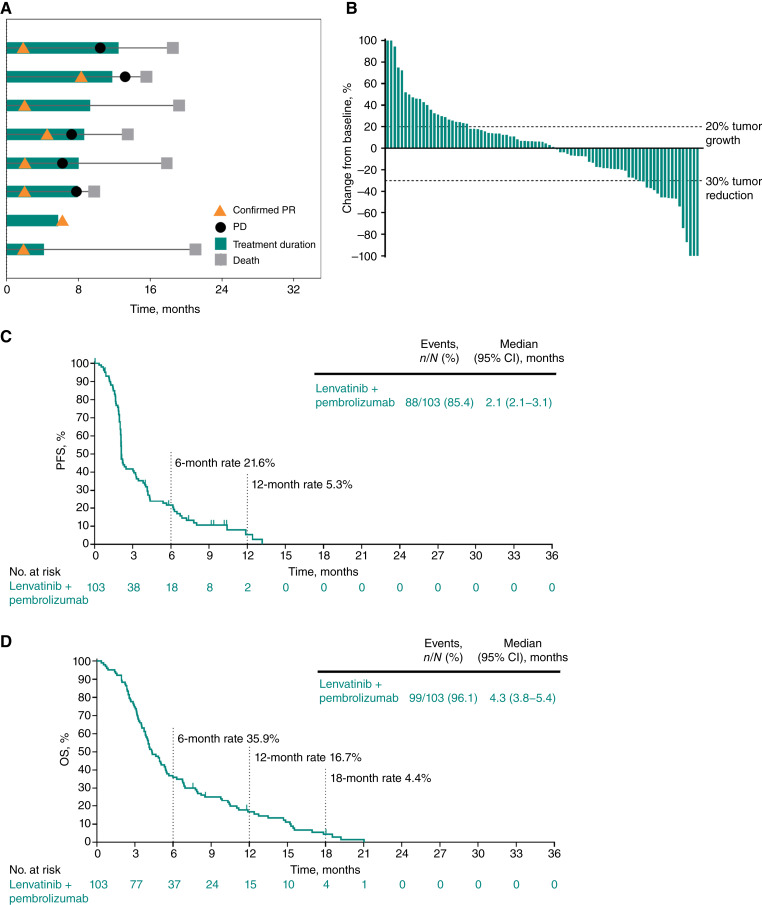
Efficacy results in cohort G, PDAC. **A,** Time on study treatment and treatment response per RECIST version 1.1 by BICR for participants with an objective response (confirmed CR or PR). **B**, Best percentage change from baseline in target lesion size per RECIST version 1.1 by BICR among participants with ≥1 postbaseline assessment. **C**, PFS per RECIST version 1.1 by BICR. **D**, OS. In **B**, percentage changes from baseline >100% are presented as 100%.

The ORR (95% CI) per RECIST version 1.1 by BICR in cohort G was 7.8% (3.4%–14.7%); eight participants (7.8%) had a PR, and no participants achieved a CR (Table 2). The DCR (95% CI) was 37.9% (28.5%–48%). ORR was similar regardless of tumor PD-L1 CPS. Among the eight participants with a response, the median DOR (range) was 5.8 (2.1+ to 8.6) months (Table 2; Fig. 4A). In the 87 participants with ≥1 postbaseline tumor assessment, 40 (46%) had a reduction in tumor size as the best percentage change from baseline in target lesions (Fig. 4B).

As of data cutoff, 88 participants (85.4%) in cohort G had experienced a PFS event. The median (95% CI) PFS was 2.1 (2.1–3.1) months, with PFS rates of 21.6% at 6 months and 5.3% at 12 months (Fig. 4C). Ninety-nine participants (96.1%) had died at the time of data cutoff. The median (95% CI) OS was 4.3 (3.8–5.4) months, with OS rates of 35.9%, 16.7%, and 4.4% at 6, 12, and 18 months, respectively (Fig. 4D).

One hundred and one participants in cohort G had tumors that were classified as non–MSI-H per local testing [74 of these participants (73.3%) had missing data]. The ORR and DOR in participants whose tumors were classified as non–MSI-H are provided in Supplementary Table S2. PFS and OS by baseline PD-L1 status are provided in Supplementary Fig. S12A and S12B.

### Safety

#### Cohort C, gastric cancer

Ninety participants (90.9%) in cohort C experienced treatment-related AEs of any grade, and 54 (54.5%) experienced grade 3 to 5 treatment-related AEs. Five participants (5.1%) died because of a treatment-related AE (esophageal perforation, *n* = 2; hemorrhage, tumor hemorrhage, and gastric perforation, *n* = 1 each; Table 3). Sixteen participants (16.2%) discontinued study treatment (either one or both study drugs) because of a treatment-related AE. Immune-mediated AEs and infusion reactions of any grade, regardless of attribution to study treatment by the investigator, occurred in 39 participants (39.4%); grade 3 to 4 immune-mediated AEs occurred in eight participants (8.1%); no participant died because of an immune-mediated AE (Supplementary Table S5). Clinically significant AEs for lenvatinib, regardless of attribution to study treatment, occurred in 77 participants (77.8%; Supplementary Table S5). Grade 3 to 5 clinically significant AEs for lenvatinib events occurred in 38 participants (38.4%), including six participants with grade 5 AEs (esophageal perforation, *n* = 2; gastric perforation, gastric hemorrhage, hemorrhage, and tumor hemorrhage, *n* = 1 each).

**Table 3.** Treatment-related AEs among all participants treated.

	Gastric cancer (cohort C) *N* = 99	BTC (cohort F) *N* = 102	PDAC (cohort G) *N* = 103
Participants with any treatment-related^a^ AE	90 (90.9)	96 (94.1)	96 (93.2)
Grade 3	44 (44.4)	58 (56.9)	53 (51.5)
Grade 4	5 (5.1)	4 (3.9)	8 (7.8)
Grade 5	5 (5.1)	0	0
Led to discontinuation of treatment	16 (16.2)	13 (12.7)	11 (10.7)

	Any grade	Grade ≥3	Any grade	Grade ≥3	Any grade	Grade ≥3
Treatment-related^a^ AEs of any grade occurring in ≥10% of participants in any cohort						
Hypertension	33 (33.3)	15 (15.2)	51 (50)	31 (30.4)	48 (46.6)	29 (28.2)
Diarrhea	24 (24.2)	2 (2)	29 (28.4)	3 (2.9)	16 (15.5)	0
Asthenia	21 (21.2)	5 (5.1)	20 (19.6)	1 (1)	16 (15.5)	2 (1.9)
Hypothyroidism	21 (21.2)	0	28 (27.5)	0	26 (25.2)	0
Fatigue	17 (17.2)	5 (5.1)	32 (31.4)	4 (3.9)	21 (20.4)	5 (4.9)
Nausea	17 (17.2)	0	23 (22.5)	1 (1)	21 (20.4)	3 (2.9)
Decreased appetite	15 (15.2)	2 (2)	18 (17.6)	1 (1)	15 (14.6)	2 (1.9)
Dysphonia	14 (14.1)	0	26 (25.5)	0	12 (11.7)	0
Increased aspartate aminotransferase	10 (10.1)	0	15 (14.7)	3 (2.9)	13 (12.6)	2 (1.9)
Palmar–plantar erythrodysaesthesia syndrome	10 (10.1)	1 (1)	11 (10.8)	0	12 (11.7)	3 (2.9)
Proteinuria	10 (10.1)	0	18 (17.6)	5 (4.9)	19 (18.4)	6 (5.8)
Pruritus	10 (10.1)	0	11 (10.8)	0	5 (4.9)	0
Vomiting	10 (10.1)	0	10 (9.8)	1 (1)	9 (8.7)	0
Mucosal inflammation	9 (9.1)	1 (1)	15 (14.7)	0	4 (3.9)	0
Stomatitis	8 (8.1)	1 (1)	12 (11.8)	1 (1)	8 (7.8)	0
Headache	6 (6.1)	0	13 (12.7)	0	10 (9.7)	1 (1)
Rash	3 (3)	0	15 (14.7)	0	13 (12.6)	1 (1)

All data are *n* (%).

a Determined by the investigator to be related to study drug.

#### Cohort F, BTC

Ninety-six participants (94.1%) in cohort F experienced treatment-related AEs of any grade. Grade 3 to 4 treatment-related AEs occurred in 62 participants (60.8%); no participant had a grade 5 treatment-related AE (Table 3). Thirteen participants (12.7%) discontinued study treatment because of a treatment-related AE. Immune-mediated AEs and infusion reactions of any grade, regardless of attribution to study treatment by the investigator, occurred in 48 participants (47.1%); grade 3 to 4 immune-mediated AEs occurred in nine participants (8.8%); none were grade 5 (Supplementary Table S6). Clinically significant AEs for lenvatinib of any grade, regardless of attribution to study treatment by the investigator, occurred in 93 participants (91.2%; Supplementary Table S6). Of these, grade 3 to 5 events occurred in 56 participants (54.9%), including three (2.9%) with grade 5 events (intestinal perforation, peritonitis, and hepatic failure, *n* = 1 each).

#### Cohort G, PDAC

Ninety-six participants (93.2%) in cohort G experienced treatment-related AEs of any grade. Grade 3 to 4 treatment-related AEs occurred in 61 participants (59.2%); no participant had a grade 5 treatment-related AE (Table 3). Eleven participants (10.7%) discontinued study treatment because of a treatment-related AE. Immune-mediated AEs and infusion reactions, regardless of attribution to study treatment by the investigator, occurred in 41 participants (39.8%); grade 3 to 4 immune-mediated AEs occurred in seven participants (6.8%); none were grade 5 (Supplementary Table S7). Clinically significant AEs for lenvatinib of any grade, regardless of attribution to study treatment by the investigator, occurred in 88 participants (85.4%), and grade 3 to 5 events occurred in 56 participants (54.4%; Supplementary Table S7). Four participants (3.9%) had grade 5 clinically significant AEs for lenvatinib (ischemic stroke, intestinal perforation, intracranial hemorrhage, and subdural hematoma, *n* = 1 each).

## Discussion

In this analysis of participants enrolled in the gastric cancer, BTC, and PDAC cohorts of the single-arm, phase II LEAP-005 study, lenvatinib plus pembrolizumab demonstrated modest antitumor activity as second- or third-line therapy and a manageable safety profile consistent with the known safety profile of this combination (9, 19). Responses were observed regardless of PD-L1 status and were generally durable. Although these results provide important information on outcomes with lenvatinib plus pembrolizumab in patients with these tumor types, considering the evolving treatment landscape, the results did not justify further investigation of this combination in phase III studies in these tumor types. Since the time of designing the LEAP-005 study, several therapies have demonstrated improved outcomes in patients with advanced disease, including several immunotherapies, and selection of therapy is increasingly being based on results from molecular testing (4–6).

In the gastric cancer cohort, which included participants with disease who progressed on two prior lines of therapy, lenvatinib plus pembrolizumab was associated with an ORR of 15.2%, median PFS of 3.5 months, and median OS of 4.4 months. This is generally comparable with currently recommended second-line or later regimens for advanced gastric cancer; although ramucirumab (anti-VEGFR2 antibody) plus paclitaxel demonstrated higher ORR of 28%, and longer median PFS of 4.4 months and median OS of 9.6 months in the phase III RAINBOW trial (37), results of the present study were similar to those previously observed with some chemotherapy regimens (associated with ORRs of 0%–29%, median PFS of 2.2–3.7 months, and median OS of 4–9.5 months; refs. 4, 38–43). There is limited evidence of the efficacy of lenvatinib monotherapy in patients with gastric cancer. As monotherapy, pembrolizumab had an ORR of 11.6% in patients with advanced gastric or GEJ cancer who had previously received ≥2 lines of treatment (44). Although the ORR observed in the current study was lower than that previously reported in a phase II study of lenvatinib plus pembrolizumab as first- or second-line therapy in participants from Japan with advanced gastric cancer [ORR (95% CI), 69% (49%–85%); ref. 30], differences in the study populations, particularly the greater number of prior therapies for participants in the present study, may have contributed to this difference. Although cross-trial comparisons should be made cautiously, it is also interesting to note that the ORR among participants with gastric cancer in LEAP-005 compares favorably with that reported for participants with previously treated gastric cancer receiving anti–PD-(L)1 therapies in other studies. For example, in the ATTRACTION-2 study, nivolumab was found to prolong OS after 3 years of follow-up versus placebo in participants with advanced gastric cancer who had received ≥2 chemotherapy regimens; the ORR among participants who received nivolumab was 11.9% (45, 46). In the JAVELIN Gastric 300 study, avelumab did not improve OS versus chemotherapy as third-line therapy for advanced gastric cancer or GEJ cancer; the ORR among participants who received avelumab was 2.2% (47). Based on prior evidence from KEYNOTE-859 that pembrolizumab may have greater benefit in patients with tumor PD-L1 CPS ≥10 versus CPS <10 in gastric cancer (31), the CPS threshold in the gastric cohort of the current study was set at CPS ≥10. In this cohort, ORR was higher in participants with tumor PD-L1 CPS ≥10 than in those with CPS <10, consistent with the findings of KEYNOTE-859 (31). MSI-H status is also an established biomarker for response to pembrolizumab; however, the potential impact of this on clinical outcomes in this study is unclear as the majority of participants in this cohort had missing MSI status (48).

Patients with advanced BTC have limited treatment options following failure of first- and second-line therapies, particularly among patients whose tumors lack an actionable biomarker (5). The current analysis reported an ORR of 17.6%, median PFS of 4.1 months, and median OS of 7.9 months in participants with BTC who had received one prior line of therapy. Although the ORR results from our trial compare favorably with those for FOLFOX (ORR, 5%), which is currently recommended as subsequent treatment for advanced BTC, PFS and OS results are comparable (median PFS of 4 months and median OS of 6.2 months with FOLFOX; refs. 5, 49). The ORR in the current analysis is numerically higher than that observed with lenvatinib monotherapy (11.5%; ref. 21) and pembrolizumab monotherapy (5.8% and 13%; ref. 11) in participants who had received ≥1 line of prior therapy, suggesting that there may be potential for clinical benefit with combination therapy. Other studies have evaluated combinations of VEGF inhibitors or lenvatinib specifically combined with anti–PD-(L)1 therapy, with or without chemotherapy, in participants with advanced BTC (50–53). Most of the participants enrolled in these trials were treatment naïve, making comparisons with the previously treated LEAP-005 population difficult; however, a phase Ib/II study in participants with advanced BTC who had received ≥1 line of prior therapy demonstrated antitumor activity with the combination of modified FLOFOX6, bevacizumab, and atezolizumab (52). Similar to prior studies of pembrolizumab monotherapy, results of the present study of lenvatinib plus pembrolizumab suggest modestly higher antitumor activity in patients with PD-L1–expressing versus PD-L1–nonexpressing BTC (11). The potential impact of MSI status on the efficacy of lenvatinib plus pembrolizumab in this cohort is unclear as all participants had missing MSI status per local testing.

There are a number of biomarker-directed therapies for BTC, such as those targeting tumors with *FGFR2* or *IDH1* alterations or HER2-positive tumors (5); thus, we performed additional exploratory biomarker analyses to further assess whether the efficacy of lenvatinib plus pembrolizumab was associated with actionable biomarkers in this tumor type. Although the number of participants who were positive for individual alterations was limited and no formal statistical testing was performed, when combining all participants with any targetable alteration, we found a trend for higher ORR among participants with targetable alterations compared with those without targetable alterations or the overall BTC cohort. It is unclear which specific alterations may be driving this higher ORR. There was a trend for higher ORR and longer PFS and OS in participants with *TP53*–wild-type tumors versus those with *TP53*-mutant tumors. This observation is in agreement with previous studies suggesting that *TP53* mutations are associated with poorer prognosis in patients with cholangiocarcinoma (54) and in patients with BTC receiving immunotherapy (55). We did not identify an association between TMB and ORR, which contrasts with prior findings that TMB is associated with response to pembrolizumab monotherapy (17, 18). There were few participants with TMB-H status in the BTC cohort (*n* = 3), which may explain the lack of association observed in this study. Additionally, the finding that Tcell_inf_GEP was not associated with ORR contrasts with prior evidence demonstrating its association with response to pembrolizumab monotherapy in advanced solid tumors (17), suggesting that it has less relevance for patients treated with lenvatinib plus pembrolizumab. Notably, we found that the *RAS* signature scores, which were determined by RNA sequencing, were higher among participants with versus without *KRAS* mutations, as determined by WES, and that these mutations were consistently negatively associated with clinical outcomes. Specifically, the *RAS* gene signature was negatively associated with PFS, and *KRAS* mutations were associated with lower ORR. These results differ from those of another study in participants with BTC receiving checkpoint inhibitors, which found a higher ORR among those with *KRAS* mutations (56). Considering the exploratory nature of the biomarker analyses in the present study, it is unclear whether this difference represents a true difference in the effects of lenvatinib plus pembrolizumab versus immune checkpoint inhibitors alone in *KRAS*-mutated BTC. Of note, *KRAS* mutations have been found to be negatively associated with prognosis in patients with certain BTC subtypes (54, 57, 58), which may explain the negative association observed in the current study in the absence of a treatment effect. Furthermore, the expression of two lenvatinib targets, *FGFR3* and *FGFR4*, showed a positive trend of association with response, suggesting that these are possible biomarkers for lenvatinib plus pembrolizumab; however, these results need to be confirmed in an independent cohort. Notably, these exploratory biomarker analyses were intended to be hypothesis generating, and the single-arm design of this study and small number of participants in biomarker subgroups limit interpretation of these results. Overall, our findings may potentially be explained by lenvatinib plus pembrolizumab combination therapy resulting in antitumor activity among participants who may have derived limited benefit from monotherapy. It remains unclear which patients with BTC may be more likely to benefit from the combination of lenvatinib plus pembrolizumab compared with monotherapy. Further research would be needed to better understand the activity of lenvatinib plus pembrolizumab in biomarker-actionable BTC.

Lenvatinib plus pembrolizumab had antitumor activity in a subset of participants with PDAC who received one or two lines of prior therapy, with an ORR of 7.8%, median PFS of 2.1 months, and median OS of 4.3 months. These results are comparable with outcomes with 5-fluorouracil–based chemotherapy regimens, which, in the absence of actionable biomarkers and depending on performance status and prior therapy, may be used as subsequent treatment for unresectable or metastatic PDAC (6). These 5-fluorouracil–based regimens have demonstrated ORRs of 1% to 16%, median PFS of 1.5 to 3.1 months, and median OS of 3.3 to 9.9 months in phase III trials with similar study populations to the current study (59). Although the outcomes in the current study are modest, it is important to note that there has been little evidence of antitumor activity with pembrolizumab monotherapy in patients with PDAC beyond those with mismatch repair–deficient/MSI-H pancreatic tumors (48). The potential impact of MSI-H status on outcomes in this study is unclear as most participants had missing MSI status. Outcomes seemed to be moderately better with lenvatinib plus pembrolizumab in participants with tumor PD-L1 CPS <1 versus CPS ≥1 in this cohort; this may be explained by prior evidence suggesting that PD-L1 expression is associated with poorer prognosis in PDAC (60). Studies of other anti–PD-(L)1–based combination therapies have also demonstrated modest antitumor activity in metastatic PDAC. Nivolumab in combination with gemcitabine and nab-paclitaxel demonstrated an ORR of 18% in participants with locally advanced or metastatic pancreatic adenocarcinoma (61), and nivolumab with or without the anti-CD40 antibody sotigalimab plus gemcitabine and nab-paclitaxel had an ORR of 50% as first-line therapy in participants with metastatic PDAC (62).

The safety profile of lenvatinib plus pembrolizumab was consistent with the known safety profiles for each treatment as monotherapy or combined therapy (9, 19). There was no evidence of worsened toxicity with the combination, which is unsurprising given the differing targets and mechanisms of action for the two therapies. Hypertension was the most commonly occurring treatment-related AE and clinically significant treatment-emergent AE for lenvatinib in each cohort. Hypertension has previously been reported to occur during lenvatinib treatment and may result from decreased VEGF signaling leading to reduced production of vasodilators (e.g., nitric oxide and prostaglandin 2) and increased production of vasoconstrictors (e.g., endothelin-1), increased vascular tone, and arterial remodeling (63). Notably, hypertension was also among the most common AEs reported with ramucirumab, an anti–VEGFR2 antibody, when combined with chemotherapy for gastric cancer in the RAINBOW trial (37). Overall, safety findings from the present study were generally consistent with other cohorts from the LEAP-005 study in participants with triple-negative breast, ovarian, and colorectal cancers (64).

In conclusion, lenvatinib plus pembrolizumab had modest antitumor activity with a manageable safety profile as a second- or third-line treatment in participants with advanced gastric cancer, BTC, or PDAC. These results contribute to the ongoing research of immunotherapy combinations for patients with these cancer types and could inform rational design of future clinical trials.

## Supplementary Material

Data Availability Statement Full version of data availability statement

Supplementary Table 1 Representativeness of the study population

Supplementary Table 2 Response and response duration in participants whose tumors were non–MSI-H

Supplementary Table 3 Response in tumor biomarker subgroups among participants with biliary tract cancer (cohort F)

Supplementary Table 4 Hypothesis testing of associations between TcellinfGEP and non TcellinfGEP consensus signatures, and clinical outcomes in participants with biliary tract cancer (cohort F)

Supplementary Table 5 Immune-mediated adverse events and clinically significant adverse events for lenvatinib by grade in cohort C

Supplementary Table 6 Immune-mediated adverse events and clinically significant adverse events for lenvatinib by grade in cohort F

Supplementary Table 7 Immune-mediated adverse events and clinically significant adverse events for lenvatinib by grade in cohort G

Supplementary Figure 1 Oncoprint from central WES in participants with biliary tract cancer (cohort F)

Supplementary Figure 2 PFS and OS by baseline PD-L1 status in participants with gastric cancer (cohort C)

Supplementary Figure 3 PFS and OS by baseline PD-L1 status in participants with biliary tract cancer (cohort F)

Supplementary Figure 4 PFS and OS by TP53 in participants with biliary tract cancer (cohort F)

Supplementary Figure 5 PFS and OS by KRAS in participants with biliary tract cancer (cohort F)

Supplementary Figure 6 Association between TMB and objective response in participants with biliary tract cancer (cohort F)

Supplementary Figure 7 PFS and OS by TcellinfGEP in participants with biliary tract cancer (cohort F)

Supplementary Figure 8 Association between TcellinfGEP and objective response in participants with biliary tract cancer (cohort F)

Supplementary Figure 9 Association between RAS and objective response in participants with biliary tract cancer (cohort F)

Supplementary Figure 10 RAS signature scores by tumor KRAS mutation status in participants with biliary tract cancer (cohort F)

Supplementary Figure 11 Association between FGFR single gene expression and objective response in participants with biliary tract cancer (cohort F)

Supplementary Figure 12 PFS and OS by baseline PD-L1 status in participants with pancreatic ductal adenocarcinoma (cohort G)

## Data Availability

Merck Sharp & Dohme LLC, Rahway, NJ (MSD) is committed to providing qualified scientific researchers access to anonymized data and clinical study reports from the company’s clinical trials for the purpose of conducting legitimate scientific research. The MSD data-sharing website (available at https://externaldatasharing-msd.com/) outlines the process and requirements for submitting a data request. Data will be made available for request after product approval in the United States and European Union or after product development is discontinued. There are circumstances that may prevent MSD from sharing requested data, including country- or region-specific regulations. Further details can be found in the Supplementary Material.
