# Supplementary material for: Lenvatinib plus Pembrolizumab for Patients with Previously Treated Advanced Gastric, Biliary Tract, or Pancreatic Cancer: Results from the Phase II LEAP-005 Study
Source: Cancer Res Commun. 2026 Mar 26;6(3):673–86. doi: 10.1158/2767-9764.CRC-26-0018 (PMC13018779; doi:10.1158/2767-9764.CRC-26-0018)
Supplement: Supplementary Table 1 — Representativeness of the study population [file crc-26-0018_supplementary_table_1_suppst1.docx]

**Supplementary Table 1.** Representativeness of the study population.

| **Cancer type(s)/subtypes/stage(s)/ condition** | **Biliary tract cancer** | **Gastric cancer** | **Pancreatic cancer** |
| --- | --- | --- | --- |
| Considerations related to |  |  |  |
| Sex | Incidence rates are generally balanced between the sexes | Globally, the incidence of gastric cancer is approximately twice as high in men as in women | In 2020, the proportion of new cases of pancreatic cancer globally was 53% in men and 47% in women |
| Age | Among patients in the United States, median age at the time of diagnosis is 71 years; 98% are 40 years or older | Incidence rates increase with age; gastric cancer is rare among patients aged younger than 45 years | Incidence of pancreatic cancer increases with age; the highest proportion of new cases are reported among patients aged 65–74 years (31.6%) |
| Race/ethnicity | In the United States, BTC is observed across all ethnicities | In the United States, incidence of gastric cancer is highest among African Americans and Asian patients | In the United States, pancreatic cancer is observed across all ethnicities |
| Geography | Incidence of BTC is higher in the Asia Pacific region and South America than in European and North American countries | Incidence rates are highest in East Asia and Eastern Europe | Incidence rates are highest in Europe, North America, and Australia/New Zealand |
| Overall representativeness of this study | Participants in each of these cohorts were enrolled globally. The demographic and baseline characteristics in our study are broadly consistent with that reported in the literature. | | |
