# Supplementary material for: Lenvatinib plus Pembrolizumab for Patients with Previously Treated Advanced Gastric, Biliary Tract, or Pancreatic Cancer: Results from the Phase II LEAP-005 Study
Source: Cancer Res Commun. 2026 Mar 26;6(3):673–86. doi: 10.1158/2767-9764.CRC-26-0018 (PMC13018779; doi:10.1158/2767-9764.CRC-26-0018)
Supplement: Supplementary Table 2 — Response and response duration in participants whose tumors were non–MSI-H [file crc-26-0018_supplementary_table_2_suppst2.docx]

## **Supplementary Table 2.** Response and response duration in participants whose tumors were non‒MSI-H.

|  | Gastric cancer (cohort C)  N = 97^a^ | **Biliary tract cancer (cohort F)**  **N = 102^b^** | **Pancreatic ductal adenocarcinoma (cohort G)**  **N = 101^c^** |
| --- | --- | --- | --- |
| ORR (95% CI), % | 15.5 (8.9‒24.2) | 17.6 (10.8‒26.4) | 7.9 (3.5‒15.0) |
| DOR,^d^ median (range), mo | 8.3 (3.4–16.2+) | 6.2 (2.7–19.6+) | 5.8 (2.1+–8.6) |

“+” indicates there was no progressive disease at the time of last disease assessment.

^a^Non-MSI-H status based on the following, per local testing: MSI-L, n = 3; MSS, n = 10; and missing MSI status, n = 84.

^b^Non-MSI-H status based on the following, per local testing: missing MSI status, n = 102. Based on WES, per central testing: MSI-H, n = 1.

^c^Non-MSI-H status based on the following, per local testing: MSI-L, n = 6; MSS, n = 21; and missing MSI status, n = 74.

^d^Based on Kaplan-Meier estimate.
