# Supplementary material for: Lenvatinib plus Pembrolizumab for Patients with Previously Treated Advanced Gastric, Biliary Tract, or Pancreatic Cancer: Results from the Phase II LEAP-005 Study
Source: Cancer Res Commun. 2026 Mar 26;6(3):673–86. doi: 10.1158/2767-9764.CRC-26-0018 (PMC13018779; doi:10.1158/2767-9764.CRC-26-0018)
Supplement: Supplementary Table 3 — Response in tumor biomarker subgroups among participants with biliary tract cancer (cohort F) [file crc-26-0018_supplementary_table_3_suppst3.docx]

## Supplementary Table 3. Response in tumor biomarker subgroups among participants with biliary tract cancer (cohort F).

| **Subgroup** | **n** | **ORR (95% CI),^a^ %** |
| --- | --- | --- |
| **Whole-exome sequencing data available** | | |
| Yes | 83 | 15.7 (8.6–25.3) |
| No | 19 | 26.3 (9.1–51.2) |
| **TP53** | | |
| Mutant | 35 | 5.7 (0.7–19.2) |
| Wild type | 48 | 22.9 (12.0–37.3) |
| **KRAS** | | |
| Mutant | 14 | 7.1 (0.2–33.9) |
| Wild type | 69 | 17.4 (9.3–28.4) |
| **Targetable alterations^b^** |  |  |
| Yes | 14 | 42.9 (17.7–71.1) |
| No | 69 | 10.1 (4.2–19.8) |
| **Tcell_inf_GEP^c^** | | |
| <1^st^ tertile | 25 | 16.0 (4.5–36.1) |
| ≥1^st^ tertile | 49 | 18.4 (8.8–32.0) |

^a^95% CIs for ORR were estimated using the Clopper and Pearson method.

^b^Includes participants who had tumors with *FGFR2* fusion, *HER2* amplification, *IDH1* mutation, microsatellite instability-high status, *NTRK* fusion, or tumor mutational burden-high status.

^c^Cutoff for first tertile was a score of −0.5577.
