# Supplementary material for: Lenvatinib plus Pembrolizumab for Patients with Previously Treated Advanced Gastric, Biliary Tract, or Pancreatic Cancer: Results from the Phase II LEAP-005 Study
Source: Cancer Res Commun. 2026 Mar 26;6(3):673–86. doi: 10.1158/2767-9764.CRC-26-0018 (PMC13018779; doi:10.1158/2767-9764.CRC-26-0018)
Supplement: Supplementary Table 4 — Hypothesis testing of associations between TcellinfGEP and non TcellinfGEP consensus signatures, and clinical outcomes in participants with biliary tract cancer (cohort F) [file crc-26-0018_supplementary_table_4_suppst4.docx]

## Supplementary Table 4. Hypothesis testing of associations between Tcell_inf_GEP and non‑Tcell_inf_GEP consensus signatures, and clinical outcomes in participants with biliary tract cancer (cohort F).

| **Gene Signature** | ***P* value^a^**  **n = 74^b^** | | |
| --- | --- | --- | --- |
|  | **ORR** | **PFS** | **OS** |
| **Tcell_inf_GEP** | 0.433 | 0.295 | 0.360 |
| **Non-Tcell_inf_GEP consensus signatures without Tcell_inf_GEP adjustment** | | | |
| Angiogenesis | 0.911 | 0.813 | 0.935 |
| mMDSC | 0.911 | 0.813 | 0.935 |
| gMDSC | 0.911 | 0.813 | 0.935 |
| Glycolysis | 0.911 | 0.813 | 0.935 |
| Hypoxia | 0.794 | 0.813 | 0.935 |
| MYC | 0.911 | 0.813 | 0.935 |
| Proliferation | 0.911 | 0.813 | 0.935 |
| *RAS* | 0.721 | **0.062**^c^ | 0.282 |
| Stroma/EMT/TGFβ | 0.911 | 0.813 | 0.935 |
| WNT | 0.911 | 0.169 | 0.935 |
| MVD | 0.911 | 0.813 | 0.935 |
| **Non-Tcell_inf_GEP consensus signatures after Tcell_inf_GEP adjustment** | | | |
| Angiogenesis | 0.886 | 0.781 | 0.956 |
| mMDSC | 0.886 | 0.781 | 0.956 |
| gMDSC | 0.886 | 0.781 | 0.956 |
| Glycolysis | 0.886 | 0.781 | 0.956 |
| Hypoxia | 0.715 | 0.781 | 0.956 |
| MYC | 0.886 | 0.781 | 0.956 |
| Proliferation | 0.886 | 0.781 | 0.956 |
| *RAS* | 0.715 | **0.069**^c^ | 0.301 |
| Stroma/EMT/TGFβ | 0.886 | 0.781 | 0.956 |
| WNT | 0.886 | 0.180 | 0.956 |
| MVD | 0.886 | 0.781 | 0.956 |

EMT, epithelial-mesenchymal transition; gMDSC, granulocytic myeloid-derived suppressor cells; mMDSC, monocytic myeloid-derived suppressor cells; MVD, microvessel density; TGF, transforming growth factor.

^a^ORR was assessed using logistic regression model and PFS and OS were assessed using Cox model. All models were adjusted for ECOG performance status. Bolded *P* values indicate nominal statistical significance for Tcell_inf_GEP analyses with α = 0.05; and multiplicity-adjusted statistical significance with α = 0.1 for non-Tcell_inf_GEP analyses (adjusted for multiplicity using Hochberg step-up procedure, tested as 1 family of 11 hypotheses).

^b^Number of participants with evaluable RNA sequencing data for analysis.

^c^Negative association between *RAS* and PFS, regardless of Tcell_inf_GEP adjustment.
