# Supplementary material for: Lenvatinib plus Pembrolizumab for Patients with Previously Treated Advanced Gastric, Biliary Tract, or Pancreatic Cancer: Results from the Phase II LEAP-005 Study
Source: Cancer Res Commun. 2026 Mar 26;6(3):673–86. doi: 10.1158/2767-9764.CRC-26-0018 (PMC13018779; doi:10.1158/2767-9764.CRC-26-0018)
Supplement: Supplementary Table 5 — Immune-mediated adverse events and clinically significant adverse events for lenvatinib by grade in cohort C [file crc-26-0018_supplementary_table_5_suppst5.docx]

## Supplementary Table 5. Immune-mediated adverse events and clinically significant adverse events for lenvatinib by grade in cohort C.

| **Adverse event** | Gastric cancer  (cohort C)  **N = 99** | |
| --- | --- | --- |
|  | **Any grade** | **Grade ≥3** |
| Participants with any immune-mediated AE or infusion reaction^a^ | 39 (39.4) | 8 (8.1) |
| Hypothyroidism | 26 (26.3) | 0 |
| Hyperthyroidism | 6 (6.1) | 1 (1.0) |
| Colitis | 2 (2.0) | 0 |
| Hypophysitis | 2 (2.0) | 2 (2.0) |
| Pancreatitis | 2 (2.0) | 2 (2.0) |
| Pneumonitis | 2 (2.0) | 2 (2.0) |
| Thyroiditis | 1 (1.0) | 0 |
| Adrenal insufficiency | 1 (1.0) | 1 (1.0) |
| Myositis | 1 (1.0) | 1 (1.0) |
| Encephalitis | 0 | 0 |
| Infusion reaction | 0 | 0 |
| Myocarditis | 0 | 0 |
| Nephritis | 0 | 0 |
| Hepatitis | 0 | 0 |
| Severe skin reaction | 0 | 0 |
| Vasculitis | 0 | 0 |
| Myasthenic syndrome | 0 | 0 |
| Participants with any clinically significant AEs for lenvatinib^b^ | 77 (77.8) | 38 (38.4) |
| Hypertension | 37 (37.4) | 16 (16.2) |
| Hypothyroidism | 26 (26.3) | 0 |
| Hepatotoxicity | 34 (34.3) | 14 (14.1) |
| Proteinuria | 13 (13.1) | 0 |
| Hemorrhage | 26 (26.3) | 7 (7.1)^c^ |
| Palmar-plantar erythrodysesthesia syndrome | 10 (10.1) | 1 (1.0) |
| Gastrointestinal perforation | 3 (3.0) | 3 (3.0)^d^ |
| Hypocalcemia | 3 (3.0) | 0 |
| Renal event | 4 (4.0) | 3 (3.0) |
| Arterial thromboembolic event | 3 (3.0) | 2 (2.0) |
| Cardiac dysfunction | 1 (1.0) | 1 (1.0) |
| Fistula formation | 0 | 0 |
| QT prolongation | 0 | 0 |
| Posterior reversible encephalopathy syndrome | 0 | 0 |

^a^Immune-mediated AEs and infusion reactions were based on a list of preferred terms intended to capture known risks of pembrolizumab and were considered regardless of attribution to study treatment by the investigator.

^b^Clinically significant AEs for lenvatinib are based on a list of terms specified by the sponsor and considered regardless of attribution to study treatment by the investigator. Related terms are included in the preferred terms listed.

^c^3 participants had grade 5 events of gastric hemorrhage, hemorrhage, and tumor hemorrhage (n = 1 each).

^d^2 participants had grade 5 esophageal perforation and 1 participant had grade 5 gastric perforation.
