# Supplementary material for: Lenvatinib plus Pembrolizumab for Patients with Previously Treated Advanced Gastric, Biliary Tract, or Pancreatic Cancer: Results from the Phase II LEAP-005 Study
Source: Cancer Res Commun. 2026 Mar 26;6(3):673–86. doi: 10.1158/2767-9764.CRC-26-0018 (PMC13018779; doi:10.1158/2767-9764.CRC-26-0018)
Supplement: Supplementary Table 6 — Immune-mediated adverse events and clinically significant adverse events for lenvatinib by grade in cohort F [file crc-26-0018_supplementary_table_6_suppst6.docx]

## Supplementary Table 6. Immune-mediated adverse events and clinically significant adverse events for lenvatinib by grade in cohort F.

| **Adverse event** | **Biliary tract cancer (cohort F)**  **N = 102** | |
| --- | --- | --- |
|  | **Any grade** | **Grade ≥3** |
| Participants with any immune-mediated AE or infusion reaction^a^ | 48 (47.1) | 9 (8.8) |
| Hypothyroidism | 34 (33.3) | 0 |
| Hyperthyroidism | 6 (5.9) | 0 |
| Colitis | 4 (3.9) | 2 (2.0) |
| Hypophysitis | 0 | 0 |
| Pancreatitis | 0 | 0 |
| Pneumonitis | 0 | 0 |
| Thyroiditis | 1 (1.0) | 0 |
| Adrenal insufficiency | 3 (2.9) | 1 (1.0) |
| Myositis | 0 | 0 |
| Encephalitis | 1 (1.0) | 1 (1.0) |
| Infusion reaction | 2 (2.0) | 0 |
| Myocarditis | 1 (1.0) | 1 (1.0) |
| Nephritis | 0 | 0 |
| Hepatitis | 5 (4.9) | 1 (1.0) |
| Severe skin reaction | 3 (2.9) | 3 (2.9) |
| Vasculitis | 1 (1.0) | 0 |
| Myasthenic syndrome | 0 | 0 |
| Participants with any clinically significant AE for lenvatinib^b^ | 93 (91.2) | 56 (54.9) |
| Hypertension | 60 (58.8) | 35 (34.3) |
| Hypothyroidism | 34 (33.3) | 0 |
| Hepatotoxicity | 49 (48.0) | 20 (19.6)^c^ |
| Proteinuria | 22 (21.6) | 5 (4.9) |
| Hemorrhage | 20 (19.6) | 5 (4.9) |
| Palmar-plantar erythrodysesthesia syndrome | 13 (12.7) | 0 |
| Gastrointestinal perforation | 8 (7.8) | 6 (5.9)^d^ |
| Hypocalcemia | 2 (2.0) | 0 |
| Renal event | 6 (5.9) | 4 (3.9) |
| Arterial thromboembolic event | 1 (1.0) | 0 |
| Cardiac dysfunction | 2 (2.0) | 2 (2.0) |
| Fistula formation | 3 (2.9) | 0 |
| QT prolongation | 0 | 0 |
| Posterior reversible encephalopathy syndrome | 0 | 0 |

^a^Immune-mediated AEs and infusion reactions were based on a list of preferred terms intended to capture known risks of pembrolizumab and were considered regardless of attribution to study treatment by the investigator.

^b^Clinically significant AEs for lenvatinib are based on a list of terms specified by the sponsor and considered regardless of attribution to study treatment by the investigator. Related terms are included in the preferred terms listed.

^c^1 participant had grade 5 hepatic failure.

^d^1 participant had grade 5 intestinal perforation and 1 participant had grade 5 peritonitis.
