# Supplementary material for: Lenvatinib plus Pembrolizumab for Patients with Previously Treated Advanced Gastric, Biliary Tract, or Pancreatic Cancer: Results from the Phase II LEAP-005 Study
Source: Cancer Res Commun. 2026 Mar 26;6(3):673–86. doi: 10.1158/2767-9764.CRC-26-0018 (PMC13018779; doi:10.1158/2767-9764.CRC-26-0018)
Supplement: Supplementary Table 7 — Immune-mediated adverse events and clinically significant adverse events for lenvatinib by grade in cohort G [file crc-26-0018_supplementary_table_7_suppst7.docx]

## Supplementary Table 7. Immune-mediated adverse events and clinically significant adverse events for lenvatinib by grade in cohort G.

| **Adverse event** | **Pancreatic ductal adenocarcinoma  (cohort G)**  **N = 103** | |
| --- | --- | --- |
|  | **Any grade** | **Grade ≥3** |
| Participants with any immune-mediated AE or infusion reaction^a^ | 41 (39.8) | 7 (6.8) |
| Hypothyroidism | 28 (27.2) | 0 |
| Hyperthyroidism | 3 (2.9) | 0 |
| Colitis | 2 (1.9) | 2 (1.9) |
| Hypophysitis | 1 (1.0) | 0 |
| Pancreatitis | 0 | 0 |
| Pneumonitis | 0 | 0 |
| Thyroiditis | 2 (1.9) | 0 |
| Adrenal insufficiency | 0 | 0 |
| Myositis | 1 (1.0) | 1 (1.0) |
| Encephalitis | 0 | 0 |
| Infusion reactions | 2 (1.9) | 0 |
| Myocarditis | 1 (1.0) | 1 (1.0) |
| Nephritis | 1 (1.0) | 1 (1.0) |
| Hepatitis | 1 (1.0) | 1 (1.0) |
| Severe skin reaction | 1 (1.0) | 1 (1.0) |
| Vasculitis | 0 | 0 |
| Myasthenic syndrome | 1 (1.0) | 1 (1.0) |
| Participants with any clinically significant AE for lenvatinib^b^ | 88 (85.4) | 56 (54.4) |
| Hypertension | 55 (53.4) | 32 (31.1) |
| Hypothyroidism | 28 (27.2) | 0 |
| Hepatotoxicity | 35 (34.0) | 11 (10.7) |
| Proteinuria | 23 (22.3) | 7 (6.8) |
| Hemorrhage | 16 (15.5) | 4 (3.9)^c^ |
| Palmar-plantar erythrodysesthesia syndrome | 13 (12.6) | 3 (2.9) |
| Gastrointestinal perforation | 5 (4.9) | 4 (3.9)^d^ |
| Hypocalcemia | 7 (6.8) | 0 |
| Renal event | 4 (3.9) | 2 (1.9) |
| Arterial thromboembolic event | 5 (4.9) | 4 (3.9)^e^ |
| Cardiac dysfunction | 0 | 0 |
| Fistula formation | 1 (1.0) | 0 |
| QT prolongation | 2 (1.9) | 0 |
| Posterior reversible encephalopathy syndrome | 1 (1.0) | 1 (1.0) |

^a^Immune-mediated AEs and infusion reactions were based on a list of preferred terms intended to capture known risks of pembrolizumab and were considered regardless of attribution to study treatment by the investigator.

^b^Clinically significant AEs for lenvatinib are based on a list of terms specified by the sponsor and considered regardless of attribution to study treatment by the investigator. Related terms are included in the preferred terms listed.

^c^2 participants had grade 5 events of intracranial hemorrhage and subdural hematoma (n = 1 each).

^d^1 participant had grade 5 intestinal perforation.

^e^1 participant had grade 5 ischemic stroke.
