# Supplementary material for: Lenvatinib plus Pembrolizumab for Patients with Previously Treated Advanced Gastric, Biliary Tract, or Pancreatic Cancer: Results from the Phase II LEAP-005 Study
Source: Cancer Res Commun. 2026 Mar 26;6(3):673–86. doi: 10.1158/2767-9764.CRC-26-0018 (PMC13018779; doi:10.1158/2767-9764.CRC-26-0018)
Supplement: Supplementary Figure 1 — Oncoprint from central WES in participants with biliary tract cancer (cohort F) [file crc-26-0018_supplementary_figure_1_suppsf1.pdf]

**Supplementary Figure 1.**

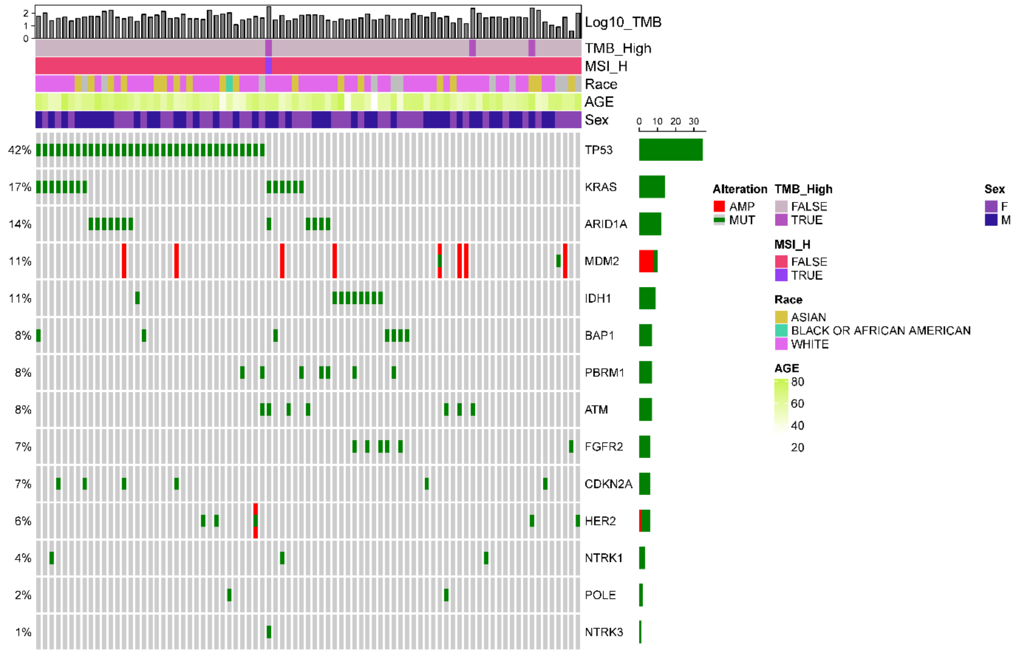


Oncoprint (n = 83) obtained from central WES performed on formalin-fixed paraffin-embedded sections of pretreatment tumor samples and matched normal (blood cell) samples using ACE Cancer Exome (Personalis Inc., Menlo Park, CA) with average coverage of 200 (range, 13-475) as previously described (33,36). WES reads were aligned to Genome Reference Consortium Human Build 37 by using Burrows-Wheeler Aligner MEM, followed by preprocessing steps that included duplicate marking, indel realignment, and base recalibration using Picard (v.1.114) and generation of analysis-ready BAM files using the Genome Analysis Toolkit (v.2). Thereafter, somatic SNV calls were generated by comparing BAM files from tumor and matched normal samples using default parameters in MuTect. MuTect-called SNVs present in the Single Nucleotide Polymorphism Database (v.141) but not in the Catalogue of Somatic Mutations in Cancer (v.68) were filtered out. SNVs with mutant reads of fewer than 4 in tumor samples were also eliminated. MuTect2 was further used to comprehensively characterize insertion or deletion/spliced mutations. Tumor mutational burden was calculated as non-synonymous mutations across all genes.

Microsatellite instability was assessed by next generation sequencing. Race, age, and sex are also shown. BAM, binary alignment map; SNV, single nucleotide variant.
