# Supplementary material for: Lenvatinib plus Pembrolizumab for Patients with Previously Treated Advanced Gastric, Biliary Tract, or Pancreatic Cancer: Results from the Phase II LEAP-005 Study
Source: Cancer Res Commun. 2026 Mar 26;6(3):673–86. doi: 10.1158/2767-9764.CRC-26-0018 (PMC13018779; doi:10.1158/2767-9764.CRC-26-0018)
Supplement: Supplementary Figure 6 — Association between TMB and objective response in participants with biliary tract cancer (cohort F) [file crc-26-0018_supplementary_figure_6_suppsf6.pdf]

Supplementary Figure 6.

A.

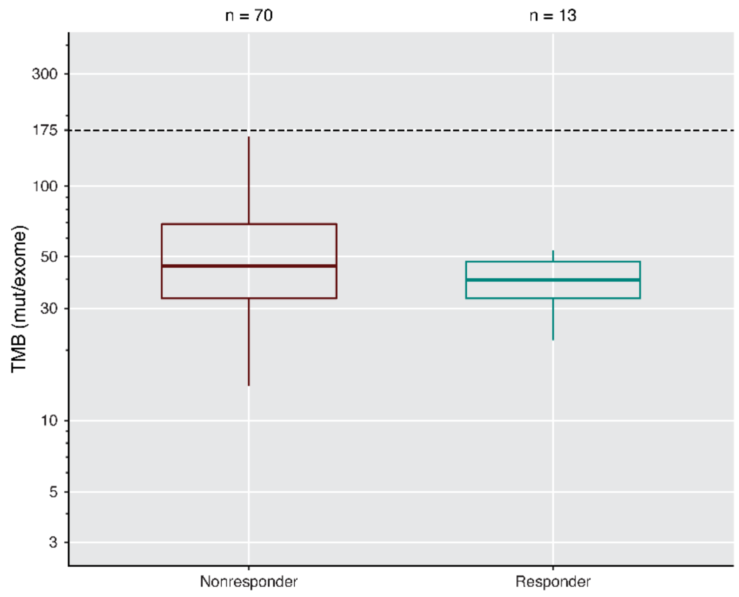

B.

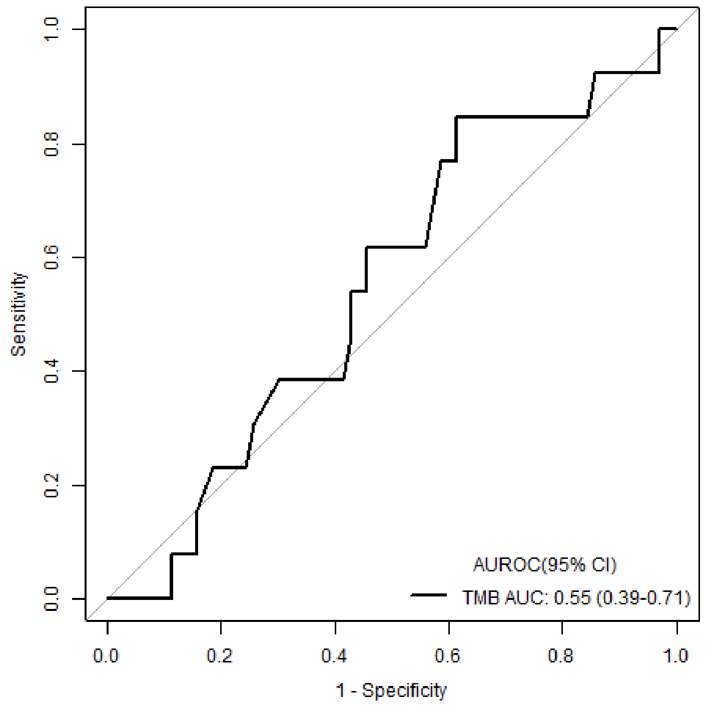

Association between TMB and objective response in participants with biliary tract cancer (cohort F). (A) TMB box plots by responder status and (B) AUROC curve for response.
