# Supplementary material for: Lenvatinib plus Pembrolizumab for Patients with Previously Treated Advanced Gastric, Biliary Tract, or Pancreatic Cancer: Results from the Phase II LEAP-005 Study
Source: Cancer Res Commun. 2026 Mar 26;6(3):673–86. doi: 10.1158/2767-9764.CRC-26-0018 (PMC13018779; doi:10.1158/2767-9764.CRC-26-0018)
Supplement: Supplementary Figure 7 — PFS and OS by TcellinfGEP in participants with biliary tract cancer (cohort F) [file crc-26-0018_supplementary_figure_7_suppsf7.pdf]

Supplementary Figure 7.

A.

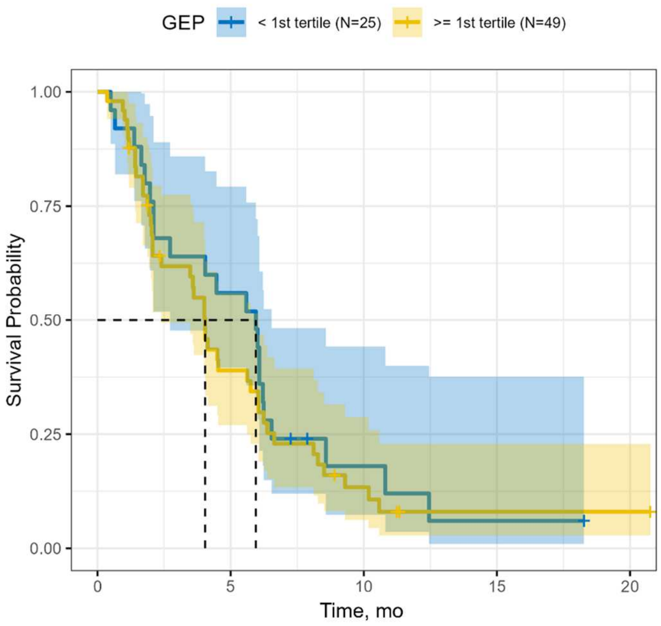

B.

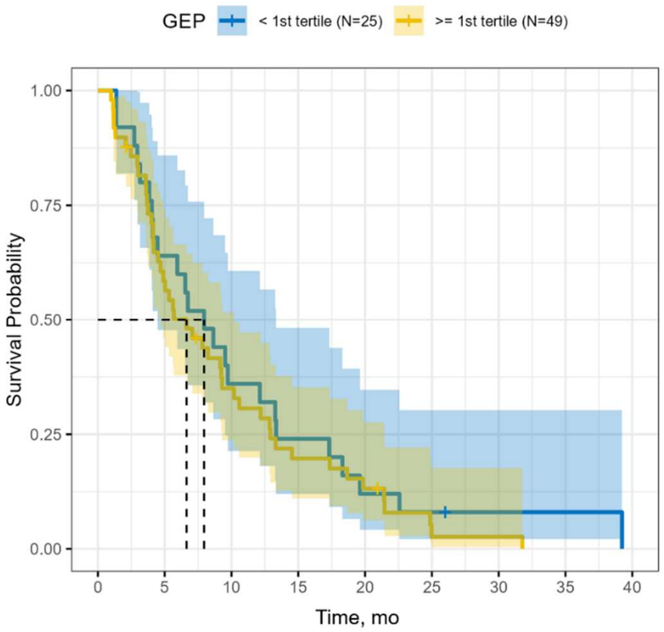

(A) Progression-free survival and (B) overall survival by $Tcell_{inf}GEP$ in participants with biliary tract cancer (cohort F). Cutoff for $Tcell_{inf}GEP$ first tertile was a score of −0.5577.
