# Supplementary material for: Lenvatinib plus Pembrolizumab for Patients with Previously Treated Advanced Gastric, Biliary Tract, or Pancreatic Cancer: Results from the Phase II LEAP-005 Study
Source: Cancer Res Commun. 2026 Mar 26;6(3):673–86. doi: 10.1158/2767-9764.CRC-26-0018 (PMC13018779; doi:10.1158/2767-9764.CRC-26-0018)
Supplement: Supplementary Figure 9 — Association between RAS and objective response in participants with biliary tract cancer (cohort F) [file crc-26-0018_supplementary_figure_9_suppsf9.pdf]

Supplementary Figure 9.

A.

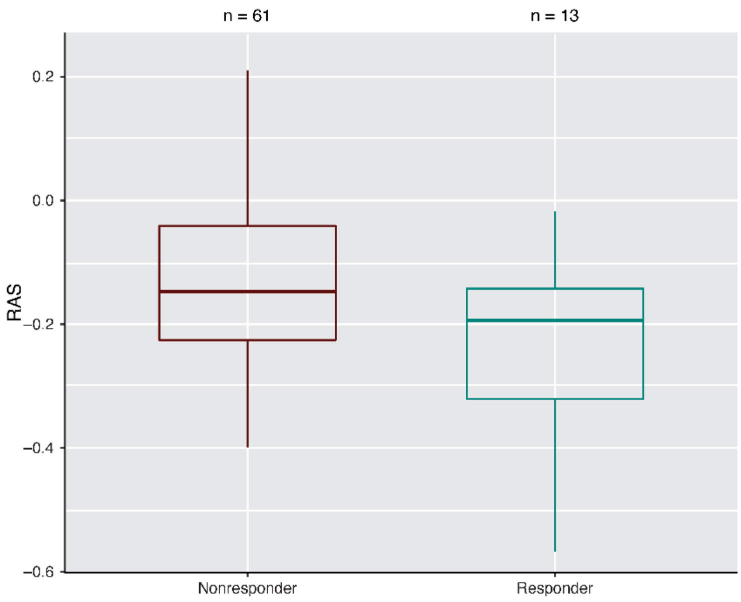

B.

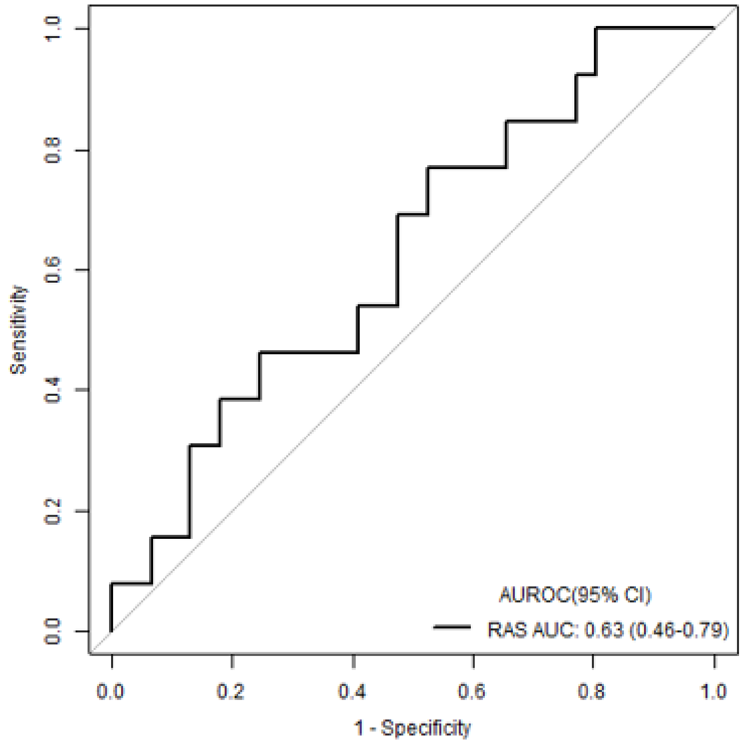

Association between *RAS* and objective response in participants with biliary tract cancer (cohort F). (A) *RAS* box plots by responder status and (B) AUROC curve for response.
