# Supplementary material for: Lenvatinib plus Pembrolizumab for Patients with Previously Treated Advanced Gastric, Biliary Tract, or Pancreatic Cancer: Results from the Phase II LEAP-005 Study
Source: Cancer Res Commun. 2026 Mar 26;6(3):673–86. doi: 10.1158/2767-9764.CRC-26-0018 (PMC13018779; doi:10.1158/2767-9764.CRC-26-0018)
Supplement: Supplementary Figure 10 — RAS signature scores by tumor KRAS mutation status in participants with biliary tract cancer (cohort F) [file crc-26-0018_supplementary_figure_10_suppsf10.pdf]

Supplementary Figure 10.

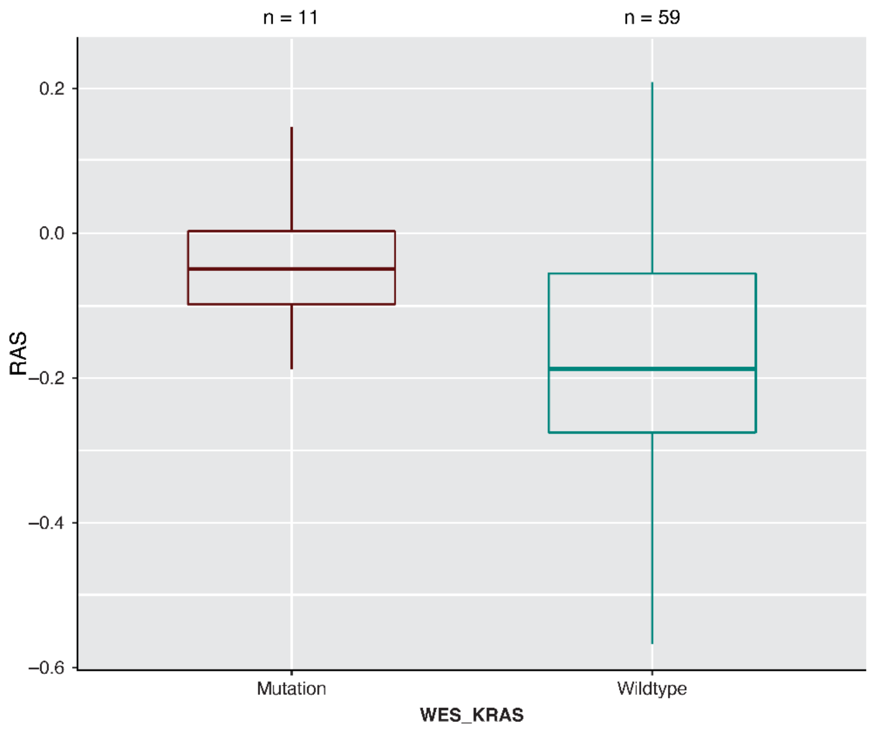

RAS signature scores, as determined by RNA sequencing, by tumor *KRAS* mutation status, as determined by WES, in participants with biliary tract cancer (cohort F).
