# Supplementary material for: Lenvatinib plus Pembrolizumab for Patients with Previously Treated Advanced Gastric, Biliary Tract, or Pancreatic Cancer: Results from the Phase II LEAP-005 Study
Source: Cancer Res Commun. 2026 Mar 26;6(3):673–86. doi: 10.1158/2767-9764.CRC-26-0018 (PMC13018779; doi:10.1158/2767-9764.CRC-26-0018)
Supplement: Supplementary Figure 11 — Association between FGFR single gene expression and objective response in participants with biliary tract cancer (cohort F) [file crc-26-0018_supplementary_figure_11_suppsf11.pdf]

Supplementary Figure 11.

A.

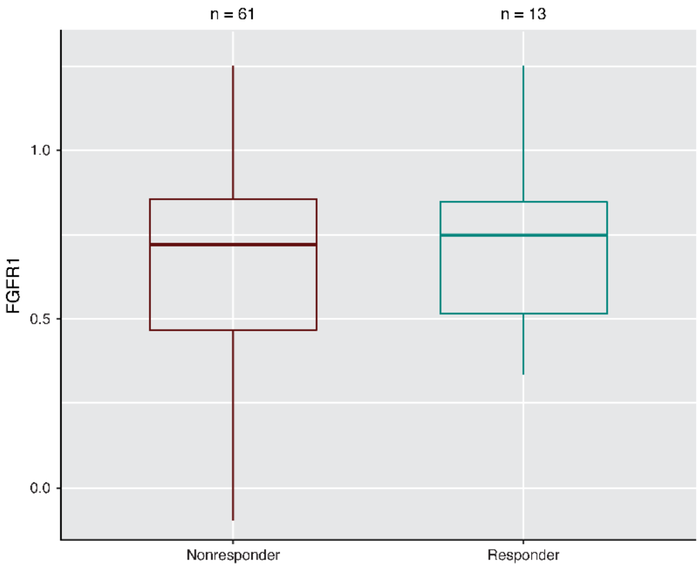

B.

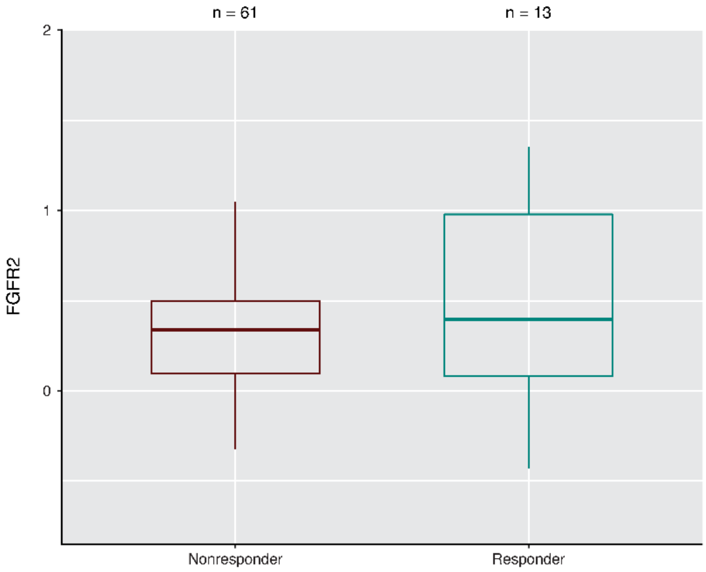

C.

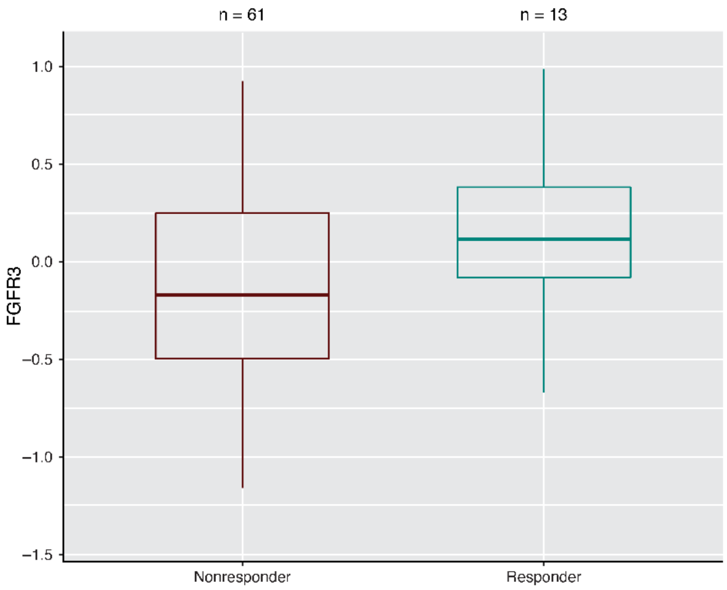

D.

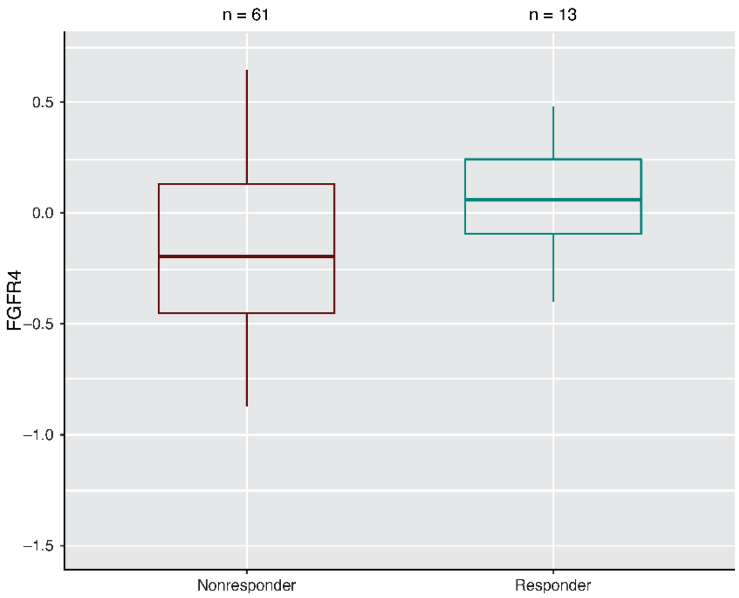

Association between *FGFR* single gene expression and objective response. (A) *FGFR1*, (B) *FGFR2*, (C) *FGFR3*, and (D) *FGFR4*.
